# Minimal Incision Scar-Less Open Umbilical Hernia Repair in Adults – Technical Aspects and Short-Term Results

**DOI:** 10.3389/fsurg.2014.00032

**Published:** 2014-09-01

**Authors:** Sanoop K. Zachariah, Najeeb Mohamed Kolathur, Mahesh Balakrishnan, Arun Joseph Parakkadath

**Affiliations:** ^1^Department of General, Laparoscopic and Gastrointestinal Surgery, Malankara Orthodox Syrian Church Medical College, Cochin, India

**Keywords:** umbilical hernia, hernia, hernioplasty, scar-less surgery, ventral hernia, visceral surgery

## Abstract

**Background:** There is no gold standard technique for umbilical hernia (UH) repair. Conventional open UH repair often produces an undesirable scar. Laparoscopic UH repair requires multiple incisions beyond the umbilicus, specialized equipments, and expensive tissue separating mesh. We describe our technique of open UH repair utilizing a small incision. The technique was derived from our experience with single incision laparoscopy. We report the technical details and short-term results.

**Methods:** This is a retrospective analysis of the first 20 patients, who underwent minimal incision scar-less open UH repair, from June 2011 to February 2014. A single intra-umbilical curved incision was used to gain access to the hernia sac. Primary suture repair was performed for defects up to 2 cm. Larger defects were repaired using an onlay mesh. In patients with a BMI of 30 kg/m^2^ or greater, onlay mesh hernioplasty was performed irrespective of the defect size.

**Results:** A total of 20 patients, 12 males and 8 females underwent the procedure. Mean age was 50 (range 29–82) years. Mean BMI was 26.27 (range 20.0–33.1) kg/m^2^. Average size of the incision was 1.96 range (1.5–2.5) cm. Mesh hernioplasty was done in nine patients. Eleven patients underwent primary suture repair alone. There were no postoperative complications associated with this technique. Average postoperative length of hospital stay was 3.9 (range 2–10) days. Mean follow-up was 29.94 months (2 weeks to 2.78 years). On follow-up there was no externally visible scar in any of the patients. There were no recurrences on final follow-up.

**Conclusion:** This technique provides a similar cosmetic effect as obtained from single port laparoscopy. It is easy to perform, safe, offers good cosmesis, does not require incisions beyond the umbilicus, and cost effective, with encouraging results on short-term follow-up. Further research is needed to assess the true potential of the technique and the long-term results.

## Introduction

An umbilical hernia (UH) is an abnormal protrusion of peritoneum through the umbilical canal, which is bounded by the linea-alba anteriorly, the umbilical fascia posteriorly, and the rectus sheath laterally on either side. The umbilicus represents one of the weak areas of the abdomen and predisposes it to hernia formation. UHs comprise 6–10% of the primary abdominal wall hernias (1). The hernia can be found centrally within the umbilicus or even laterally, superiorly, and inferiorly. UHs are classified into three types, namely, congenital, infantile, and adult types. In 90% of the cases, the adult UH is acquired (2). The formation of an UH is a complex process from an embryologic and anatomic perspective. After birth, the umbilical arteries and the vein get thrombosed and the umbilical ring contracts due to cicatrization. Impaired cicatrization coupled with the lack of elastic fibers in the obliterated umbilical vein leads to an area of potential weakness over the umbilical scar. In adults, conditions like pregnancy, obesity, and liver disease with cirrhosis, may cause the umbilical ring to stretch and reopen leading to the formation of an UH. Adult UHs are commonly seen to occur in females, with a peak incidence between the third and the fifth decades of life. They are also frequently found to be associated with obesity and liver cirrhosis.

There is no gold standard technique for UH repair. The conventional “Mayo’s technique” of open UH repair, initially described by “William Mayo” in 1895 had been the treatment of choice for more than a century and is still being performed in many parts of the world. However, this technique is becoming less popular owing to the influx of minimally invasive techniques, which utilize smaller incisions. Mayo’s technique often leaves an undesirable scar below the umbilicus (3). The standard textbooks mention than UHs can be repaired either using the suture repair technique or hernioplasty, utilizing a mesh. We have followed the same standard repair for many years. However, until now the size of the incision had not been given much importance. There is no consensus regarding the size of the incision for open UH repair. It is generally understood that open techniques require larger incisions when compared to laparoscopic surgery. However, laparoscopic UH repair requires multiple incisions beyond the umbilicus, specialized equipments, and expensive tissue separating mesh. Here, we describe our technique of open UH repair, which offers the advantages of a single small incision. The idea of placing such an incision was derived from our experience with single port laparoscopy. Our intention was to offer the patient the benefit of smaller incisions just as in laparoscopic surgery, without altering the surgical principle. The method described here shows that it is possible to perform the standard or conventional UH repair through a smaller incision. The aim of the study is to present the surgical technique and short-term results.

## Materials and Methods

This study is a retrospective analysis of the first 20 consecutive cases of minimal incision scar-less open UH repair performed by a single surgeon at the MOSC Medical College (Kolenchery, Cochin, India), for patients who presented with symptomatic UHs between June 2011 and February 2014. Data were collected from the medical records of these patients. Prior to surgery all patients underwent routine abdominal ultrasonography to confirm the presence of the hernia, rule out additional pathologies and have a preoperative assessment of the defect size. The study group comprised patients with UHs with defect size not exceeding 4 cm in diameter. The patients selected were adults, who had not undergone a previous laparotomy. All patients were advised to have a preoperative shower with a thorough cleansing of the umbilicus on the day of surgery. The patients were administered a single dose of intravenous cefuroxime at the time of induction of general anesthesia. Patients who had liver cirrhosis were initially treated by the medical gastroenterologist in order to reduce the ascites prior to surgery. The patients underwent surgery under general or regional anesthesia, depending on their preferences and also as advised by the anesthetist.

### Operative technique (Figures 1A–M)

**Figure 1:**
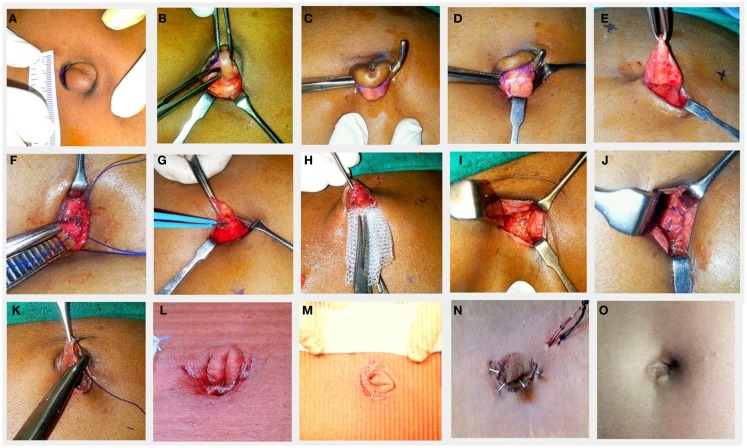
**The operative steps (A–M)**. The appearance of the umbilicus 1 year following surgery **(O)**.

The patient was placed in the supine position and the operative field was cleaned with 10% povidone iodine solution. An intra-umbilical curved skin crease incision was made as done routinely for multiport single incision laparoscopy (Figure 1A). The size of the incision depends on the diameter of the umbilicus and the size of the sac. The upper flap was held taut by an Allis forceps and the umbilical cicatrix (containing the sac) was delineated by a combination of blunt and sharp dissections. The blades of a closed Allis forceps were introduced by side of the cicatrix and after which the blades were opened (Figure 1B). By doing so all the soft tissue were separated from the sides of the cicatrix. This was repeated on the other side also. The remaining posterior portion of the umbilical cicatrix was then freed by inserting a right angled forceps, dissecting, and hooking it all around (Figure 1C). Now that the cicatrix was free all around, a small incision was made transversely over the middle of the cicatrix to open the sac (Figure 1D). The contents were identified and reduced into the peritoneal cavity after releasing any adhesions. The transverse incision was then completed all around the cicatrix. Thus, the upper flap now comprised the skin of the umbilicus above with a layer of the distal sac attached on its undersurface (Figure 1E). This is important as it prevents the skin from becoming too thin and also prevents direct contact of the skin to the mesh. The size of the defect was measured with a sterile scale and recorded. Based on this we decided on the type of repair. No artificial enlargement of the defect was performed and the edges of the sac were retained to be used as flaps for tension free closure. Using diathermy, a surrounding subcutaneous space was created by undermining it for 2 cm (suture repair) to 4 cm (mesh placement) all around depending on the size of the defect and also depending on whether or not a mesh had to be placed (Figure 1G). This was also done with a view to reduce the tension over the umbilical ring in addition to creating a space for mesh placement. Care was taken not to inadvertently incise the rectus sheath.

For defects up to 2 cm in greatest diameter, a primary suture repair was performed. This was done using number one polypropylene suture, by taking bites not more than 1 cm from the edge of the defect in a continuous fashion vertically in a way so as to just close the fascial defect and at the same time avoiding tension (Figure 1F).

For defects larger than 2 cm, a polypropylene mesh of size 8 cm × 8 cm was anchored in place over this sutured line (onlay) so as to obtain an overlap of 4 cm all around from the center of the defect (Figure 1H). The mesh needs to be rolled into the space and then spread out using a forceps. We secured the mesh by pacing the central stitch first and then subsequently at 12, 6, 3, and 9 o’clock positions, followed by a few more anchoring sutures in between (Figure 1J). For patients with a BMI of 30 kg/m^2^ or more, onlay mesh hernioplasty was performed irrespective of the size of defect.

After repair of the defect, the umbilical flap containing (the distal end of the sac) was then anchored to the surface of the mesh at the center using a 3-0 vicryl single stitch to create an inverted umbilicus in its original place (Figure 1K). The skin was the approximated using clips or 4-0 nylon sutures. A sterile padded dressing was applied and to be removed after 48 h. A suction drain was placed in the subcutaneous space only for one patient who had liver cirrhosis with ascites. The drain was removed after 48 h (Figure 1N).

Following discharge, they were advised to follow-up at 1 week, 1 month, 3 months, 6 months, and thereafter, once a year. For the present study, all these 20 patients were contacted over phone and asked to come for follow-up in the outpatient during the months of March and April 2014 to assess the scar and record the symptoms related to the surgery and to identify if there were any recurrences.

## Results

A total of 20 patients underwent minimal incision scar-less open UH repair. There were 12 males and 8 females. The mean age was 50 (range 29–82) years. The mean BMI was 26.27 (range 20.0–33.1) kg/m^2^. There were five patients who had a BMI ≥ 30 kg/m^2^ of which two patients had a defect size of more than 2 cm in greatest diameter. The mean defect size was 1.75 (range 0.5–3.4) cm. The average size of the incision was 1.96 range (1.5–2.5) cm. In three patients, the hernia was irreducible and contained omentum. Hernioplasty with onlay mesh was done in 9 patients and while 11 patients underwent primary suture repair alone. A drain was placed only in one patient, who was diagnosed to have liver cirrhosis, portal hypertension, and ascites. The mean operating time was 48 minutes and 30 seconds. The mean operating time for procedures requiring placement of mesh was 1 hour and 33 seconds while that for primary suture repair was 38 min and 38 s. The average postoperative length of hospital stay was 3.9 (range 2–10) days. The mean follow-up was 29.94 months, ranging from 2 weeks to 2.78 years. On follow-up there was no externally visible scar in any of the patients, as the scar was well hidden in the umbilicus (Figure 1O). The associated co-morbidities were systemic hypertension and type 2 diabetes mellitus (10%); chronic liver disease with portal (5%), hypertension (10%), and dyslipidemia (10%); and chronic obstructive pulmonary disease (5%). There were no postoperative complications associated with this technique. None of the patients had recurrences on short-term follow-up. None of the patients complained of pain over the surgery site on final follow-up.

## Discussion

The treatment of UH has not gained sufficient importance from the surgical fraternity in comparison with other types hernias (4). Although a common and relatively simple procedure, there is no exact protocol or universal consensus on how the repair should be carried out (3, 5).

Standard textbooks describe those small defects of <1 cm may be primarily closed with non-absorbable monofilament sutures either by a figure of eight stitch or by darning (6). Defects between 1 and 2 cm diameter may be sutured primarily just to close the defect with minimal tension. For defects larger than 2 cm, mesh repair is recommended. Schumacher et al. (7) suggested that repair with mesh should be reserved for patients with a BMI >30 kg/m^2^ and hernia orifice larger than 3 cm. A number of techniques of mesh placement have also been described and there is no prospective data demonstrating clear advantages of one technique over another. The mesh may be placed: (1) intraperitoneally, (2) in the retro-muscular space, (3) in the extra-peritoneal space, and (4) in the subcutaneous plane after closing the linea-alba vertically (onlay technique), which is the simplest. Moreover, there is no consensus on the size of the incision utilized for open repair of UHs.

In this modern era of “minimal access surgery,” the trend is toward developing surgical procedures that require significantly smaller incisions, with an attempt to reduce pain, shorten the length of hospital stay, and particularly to improve cosmesis (8). These advantages of laparoscopic surgery are attributed to the use of smaller incisions for surgical access. However, laparoscopic surgery for UHs has certain disadvantages. A recent systematic review (9), comparing the conventional open technique with the laparoscopic technique showed that the risks of the hernia recurrence with laparoscopic technique are relatively unknown and that laparoscopic repairs were associated with higher in-hospital costs and also an increased risk of intra-operative bowel injury. Laparoscopic procedures have an inherent risk of developing trocar site hernias with an overall incidence of 0–5.2% (10). Mason et al. (11) studied 71,054 patients who underwent abdominal wall hernia repair and reported that there were no differences noted between laparoscopic and open repairs in patients when the hernias were reducible, but offered lower morbidity, particularly, when hernias were complicated. The use of laparoscopic procedures may be beneficial for larger defects, especially for cosmetic considerations to be justified by the higher cost.

**Figure 2:**
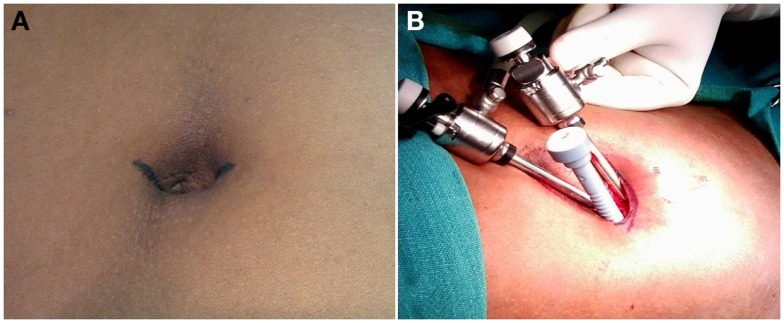
**Our technique of multiport single incision laparoscopy, where the similar type of incision is made to identify the umbilical cicatrix for placement of the ports**.

Our aim was to offer the patient the benefit of smaller incisions without altering the surgical principle. A few others too have published reports on cosmetic approach to open UH repair by making use of smaller incisions. Kurpiewski et al. (12) described a technique of using 3–3.5 cm incisions for placement of mesh in the preperitoneal space. Mislowsky et al. (13) described a scar-less suture repair technique (without mesh) for UHs <2 cm in size by utilizing a vertical intra-umbilical incision. Arslan et al. (14) reported their technique of UH repair utilizing small intra-umbilical curved incisions for hernias <4 cm in size. The idea of making a small incision and raising flaps was derived from our experience with single incision laparoscopy. In single incision laparoscopy, all the ports are positioned through a single incision located in and around or sometimes entirely through the umbilical cicatrix (Figure 2). There is only one incision that is concealed within the umbilicus and there is no visible scar. This is beneficial from a cosmetic point of view. In addition to the “scar-less” or “virtually scar-less” effect, the claimed benefits include less postoperative pain, lesser hospital stay, and earlier return to work (15, 16). The proposed advantages of our technique are outlined in Table 1.

**Table 1:** **Proposed advantages of minimal incision scar-less open umbilical hernia repair**.

	Advantages
1	Needs only a small incision, which is hidden within umbilicus
2	Avoids the need for additional incisions beyond the umbilicus
3	Specialized instruments are not required
4	Eliminates the need for expensive tissue separating meshes
5	The same technique could be modified to incorporate: sub-lay or plug techniques
6	Provides good cosmesis and virtually scar-less effect as in single incision laparoscopy
7	Can be used in patients who may be otherwise unfit for laparoscopic procedure
8	Can be performed in patients with liver cirrhosis
9	No possibility of additional trocar site hernias
10	Less expensive and cost effective

## Conclusion

The technique described here shows that it is possible to perform the standard or conventional UH repair by means of a smaller incision. The classic repair and the surgical principles are not altered. It obviates the need for special instruments and expensive meshes. The technique is easy to perform, safe, offers good cosmesis, does not need incisions beyond the umbilicus, and cost effective, with encouraging results on short-term follow-up. Further research is needed to assess the true potential of the technique and its long-term results.

## Conflict of Interest Statement

The authors declare that the research was conducted in the absence of any commercial or financial relationships that could be construed as a potential conflict of interest.
